# An Adaptive Weight Learning-Based Multitask Deep Network for Continuous Blood Pressure Estimation Using Electrocardiogram Signals

**DOI:** 10.3390/s21051595

**Published:** 2021-02-25

**Authors:** Xiaomao Fan, Hailiang Wang, Yang Zhao, Ye Li, Kwok Leung Tsui

**Affiliations:** 1 School of Computer Science, South China Normal University, Guangzhou 510631, China; xmfan@scnu.edu.cn; 2 School of Design, Hong Kong Polytechnic University, Hong Kong, China; hailiang.wang@polyu.edu.hk; 3 School of Data Science, City University of Hong Kong, Hong Kong, China; kltsui@cityu.edu.hk; 4 Shenzhen Institutes of Advanced Technology, Chinese Academy of Sciences, Shenzhen 518055, China; ye.li@siat.ac.cn

**Keywords:** continuous blood pressure, multiple tasks, weights learning, electrocardiogram

## Abstract

Estimating blood pressure via combination analysis with electrocardiogram and photoplethysmography signals has attracted growing interest in continuous monitoring patients’ health conditions. However, most wearable/portal monitoring devices generally acquire only one kind of physiological signals due to the consideration of energy cost, device weight and size, etc. In this study, a novel adaptive weight learning-based multitask deep learning framework based on single lead electrocardiogram signals is proposed for continuous blood pressure estimation. Specifically, the proposed method utilizes a 2-layer bidirectional long short-term memory network as the sharing layer, followed by three identical architectures of 2-layer fully connected networks for task-specific blood pressure estimation. To learn the importance of task-specific losses automatically, an adaptive weight learning scheme based on the trend of validation loss is proposed. Extensive experiment results on Physionet Multiparameter Intelligent Monitoring in Intensive Care (MIMIC) II waveform database demonstrate that the proposed method using electrocardiogram signals obtains estimating performance of 0.12±10.83 mmHg, 0.13±5.90 mmHg, and 0.08±6.47 mmHg for systolic blood pressure, diastolic blood pressure, and mean arterial pressure, respectively. It can meet the requirements of the British Hypertension Society standard and US Association of Advancement of Medical Instrumentation standard with a considerable margin. Combined with a wearable/portal electrocardiogram device, the proposed model can be deployed to a healthcare system to provide a long-term continuous blood pressure monitoring service, which would help to reduce the incidence of malignant complications to hypertension.

## 1. Introduction

Hypertension is a common chronic cardiovascular disease, leading to health disorders with potentially fatal complications such as stroke and heart failure [1]. According to the World Health Organization (WHO) statistic report in 2015, the prevalence of global hypertension is over 24% in the male and 20% in the female population, which shows a rising trend in recent years [2]. Unfortunately, most of individuals with hypertension are not aware of their health and the ability of this silent disease to harm them. Cuff-based blood pressure (BP) monitoring devices, mercury sphygmomanometer and electronic sphygmomanometer, are usually used to measure BP values precisely in hospital and at home. However, they are just for point-BP measurement and quite inconvenient for use in daily life, particularly for long-term continuous BP monitoring, due to the tedious repeated operation of cuff inflation and deflation [3]. Herewith, a kind of BP measurement based on physiological signals, which can be acquired by various daily used wearable/portal monitoring devices, has great significance.

Generally, BP indicators of systolic blood pressure (SBP), diastolic blood pressure (DBP), and mean arterial pressure (MAP) are utilized to evaluate a person’s BP conditions. In recent years, large amounts of continuous BP estimation methods based on electrocardiograph (ECG) and photoplethysmography (PPG) signals have been proposed to address the problem of continuous long-term BP monitoring in daily life [4,5]. Traditional BP estimation methods are based on the calculating parameters of pulse wave transit time (PWTT) and pulse wave arrival time (PWAT) through a series of operations of fiducial point determination and feature extraction [6,7,8]. For instance, literatures [6,8,9] developed BP estimation frameworks based on parameters of PWAT and PWTT, with additional vital signs, which achieved good BP estimation performance and had a considerable margin to the British Hypertension Society standard (BHS) [4] and US Association of Advancement of Medical Instrumentation standard (AAMI) [7]. Despite the advantages of efficiency and high performance, there are still two apparent existing challenges for the PWTT-based/PWAT-based methods. The first challenge is that the reliability of these methods are high depended on accuracy of fiducial point determination, which is quite sensitive to the quality of physiological signals. Another challenge is to extracting high related features from physiological signals, which require domain expertise knowledge for practitioners. To address these problems, many researchers shift their focus from PWTT-based/PWAT-based methods to cutting-edge deep learning methods.

Deep learning with the merits of end-to-end training and automatic feature engineering has been largely investigated for physiological signal analysis and achieved a big success [10,11,12]. It is also utilized for BP estimation based on physiological signals [13,14,15,16,17]. Based on ECG and PPG signals, various deep learning structures, such as back-propagation neural network [13,18], deep recurrent neural network [14], and hybrid deep neural network [17,19,20], are employed to estimate SBP, DBP, and MAP values. Experimental results demonstrated that deep learning-based methods achieved promising BP estimation results. Concerning wearable/portal devices with limited energy capability to long-term monitoring, most of them only acquiring a specific kind of physiological signals, such as ECG [16,21], PPG [22], pressure pulse wave (PPW) [23], and impedance plethysmograph (IPG) signals [24]. Even though these one-channel physiological signals can be utilized to measure the BP values, there are still existing shortcomings in practical applications of wearable/portable devices. PPG contains rich pulse fluctuation information, and the extraction of PPG waveform features can indirectly reflect the information of vascular elasticity. However, the shape of PPG waveform often has great differences between different measurement locations and different individuals; it is not suitable for BP modeling to general population. PPW directly reflects the pressure change of the outer wall of blood vessels, but its amplitude is related to the external pressure from a wearable device. The BP estimating method based on single-channel PPW signal needs to calibrate the external pressure. This method is extremely sensitive to external disturbance. The BP estimating method based on IPG arrays requires electrical sensors to be accurately placed in the center of the artery and very sensitive to human movement. A small deviation of the measurement position and human movement would lead to the instability of the accuracy of blood pressure measurement. As for ECG signals, the QRS wave of ECG reflects the information of heart ventricular contraction. With the QRS wave, the R-wave morphology can reflect the intensity of ventricular contraction and then affect cardiac ejection. It means that there would be a close correlation between ECG and BP values. Meanwhile, compared with PPG, PPW, and IPG signals, it is easier to obtain ECG signals on wearable devices, and the interference of daily activities is relatively small. Researchers [16,21,25] attempted to develop BP estimation methods only based on one-channel ECG signals with deep learning and achieved promising results. However, these methods estimate SPB, DBP, and MAP values from ECG signals directly, either without considering the commonalities and relationship among SPB, DBP and MAP estimation, or required an additional calibration by means of golden-standard BP values.

In this study, to take advantage of the commonalities and relationship among the SBP, DBP, and MAP estimation, we propose a novel calibration-free adaptive weight learning-based multitask deep learning framework, based on single lead ECG signals, to estimate continuous BP values. It is well known that a multitask network has the capability with born to share the knowledge among all the tasks. To be more specific, the proposed method utilizes a 2-layer bidirectional long short-term memory network (BiLSTM) as the sharing layer, followed by three identical task-specific 2-layer fully connected networks for SBP, DBP, and MAP estimation. The proposed method is trained with a widely used training technique called joint training. However, the BP estimation performance of the proposed multitask is sensitive to the importance of task-specific losses. Traditionally, the optimal task-specific importance is searched by a grid search method or intelligent heuristic method  [26]. However, both of them are time-consuming and high computing complexity. Herewith, we proposed an adaptive weight method to determine the task-specific importance degree based on the trend of validation loss. Extensive experiment results demonstrate that the BP estimation performance of our proposed method meets the requirements of the BHS and AAMI standards with a considerable margin. It means that a healthcare system, with collecting ECG signals, deploying our proposed model can provide a long-term continuous BP monitoring service. In general, our contributions can be summarized as follows:

• To take advantage of the relationship among SBP, DBP, and MAP, we propose a novel multitask deep learning framework based on single lead ECG signals to estimate BP values.
• To conquer the problems of time-consuming and high computing complexity on traditional weight-searching methods, we propose an adaptive weight learning-based method to determine the task-specific importance based on the trend of validation losses.
• Extensive experiment results demonstrate that the BP estimation performance of our proposed method meets the requirements of the BHS and AAMI standards with a considerable margin.

The remainder of this study is organized as follows: Section 2 describes the proposed novel BP estimation method and reference methods in detail. Section 3 demonstrates the experimental results and performance comparison with other cutting-edge methods. Section 4 discusses the performance analysis of the proposed method. Finally, this paper is concluded in Section 5.

## 2. Materials and Methods

**Figure 1:**
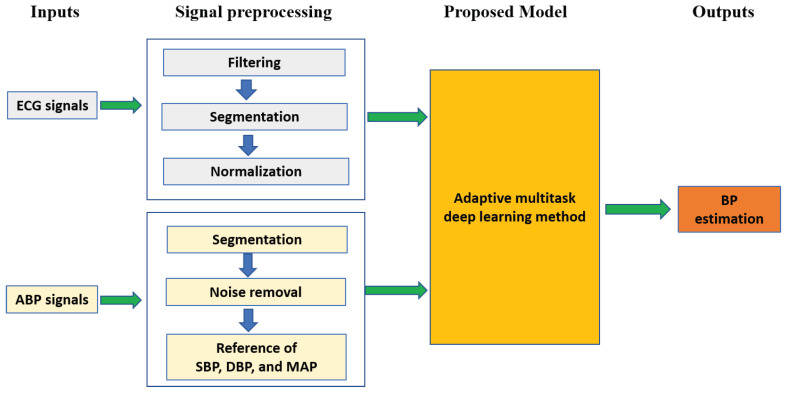
Pipeline of blood pressure estimation-based electrocardiograph (ECG) signals. The proposed multitask network is iteratively updated in the training phase by computing the root mean square loss values between estimated BP values and ground truth BP values. ABP means arterial blood pressure.

As shown in Figure 1, BP estimation mainly includes signal preprocessing, extracting ground truth BP values, and model building. In this section, the procedure of BP estimation is described in detail as follows.

### 2.1. Problem Formulation

The task of this study is to regress the BP estimation performance based on ECG signals. A multitask deep learning framework, with the merit of utilizing the relationship among SBP, DBP, and MAP estimation in born, is employed to boost the BP performance. Suppose that we have training ECG signals $X^{ecg}$ as the model inputs and corresponding reference SBP $Y^{s}$, DBP $Y^{d}$, and MAP $Y^{m}$ values extracted from synchronous arterial blood pressure (ABP) signals, the goal of each task is to learn a non-linear mapping function Φ(·) from ECG signals to corresponding SBP ${\hat{Y}}^{s}$, DBP ${\hat{Y}}^{d}$, and MAP ${\hat{Y}}^{m}$ values, which are defined to be:

(1) $$\;{\hat{Y}}^{s}=\Phi^{s}\left(X^{ecg};W^{share},W^{s}\right)$$

(2) $$\;{\hat{Y}}^{d}=\Phi^{d}\left(X^{ecg};W^{share},W^{d}\right)$$

(3) $$\;{\hat{Y}}^{m}=\Phi^{m}\left(X^{ecg};W^{share},W^{m}\right)$$

where $W^{share}$ is the parameter in the sharing layer, $W^{i\in \left\{s,d,m\right\}}$ is the parameter in the task-specific network. The naive approach to combining multiple estimation losses of SBP, DBP, and MAP would be to merely implement a weighted linear sum of the losses for each BP estimation task:

(4) $$\;L_{total}=\sum_{i\in \left\{s,d,m\right\}}\theta^{i}*L\left(Y^{i},{\hat{Y}}^{i}\right)$$

where L(·,·) is the task-specific loss function, $L_{total}$ denotes a total of BP estimation losses, $\theta^{i}$ is the importance of the loss of task-specific BP estimation, and s,d,m refer to the SBP, DBP, and MAP estimation task, respectively.

### 2.2. Preprocessing

In practice, signal preprocessing is a quite important and effective way to remove noise contamination from ECG signals, which is helpful to the subsequent BP estimation modeling. In this section, filtering with discrete wavelet transformation (DWT) [27], segmentation, and extraction of ground truth BP values from ABP signals are described as follows.

#### 2.2.1. Filtering

**Figure 2:**
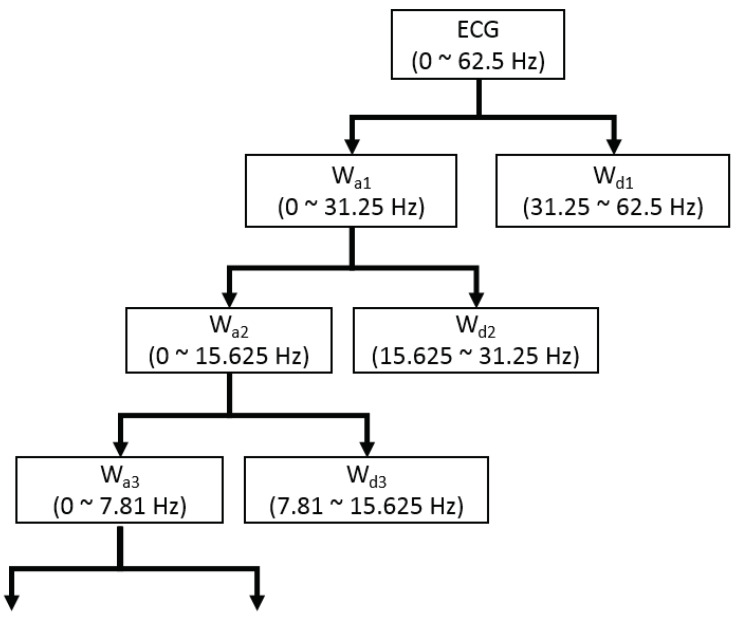
The structure of the discrete wavelet transformation (DWT) decomposition.

In the field of biomedical engineering, wavelet transform is a widely used method to remove noise from ECG signals. The wavelet transform, which has the ability to acquire time-frequency information, is a convolution of a wavelet function ψ(t) with the input signal x(t). To capture multi-resolution time-frequency information, parameters of scale σ and translation τ are employed in the wavelet function. Herewith, the continuous wavelet transform (CWT) can be defined to be:

(5) $$\;W_{x(t)}^{\psi }=\frac{1}{\sigma^{1/2}}\int x\left(t\right)\psi \left(\frac{t-\tau }{\sigma }\right)dt$$

where $W_{x(t)}^{\psi }$ is wavelet coefficients underlying input signal x(t) with the wavelet function ψ. In practice, signals to be analyzed are discrete. Therefore, DWT, obtained from CWT by utilizing discrete steps for scale and translation, is utilized to process such discrete signals. The DWT can be defined by sampling at discrete intervals:

(6) $$\;W_{x(n)}^{\psi }=\frac{1}{\sigma^{1/2}}\sum x\left(n\right)\psi_{j,k}\left(\frac{n-k\tau }{\sigma }\right)$$

where *j* refers to the level of resolution and *k* refers to the level of translation. In this study, the wavelet function ψ, also called mother wavelet, utilizes Daubechies 8 (db8) [28]. A signal is decomposed by the DWT into two parts of low frequency and high frequency, which are also known as approximate coefficients and detail coefficients. The low frequency signal is continuously downsampled by a factor of two to acquire the successive approximation coefficients, which is presented in Figure 2. Since the ECG signals analyzed in this study are sampled at 125 Hz and the useful frequency range of ECG signals is from 0.5 Hz to 45 Hz [29], it is reasonable to decompose the DWT into eight levels based on Nquist sampling theorem [30]. The frequency of the last-layer approximate coefficients is in the range of 0 to 0.24 Hz. By setting zeros of the 8-th approximate coefficients and the first detail coefficients, the ECG signals can be reconstructed by the inverse DWT, which  removes the high frequency noise and baseline wandering. Due to the frequency of reconstructed ECG signals ranging from 0.24 Hz to 31.25 Hz, we downsample the reconstructed ECG signals from 125 Hz to 100 Hz for more convenient configuration of the proposed network.

#### 2.2.2. Segmentation

According to the requirement of the proposed method, every ECG signal has to be cropped into segments with fixed length. Meanwhile, the corresponding synchronized ABP signals are also cropped into segments with the same fixed length as ECG segments for extraction of ground truth BP values. In this study, the fixed length of signals employs a time duration of 10 s, which is widely employed in deep learning approaches [10,12]. To mitigate the different amplitudes of ECG signals, the min-max normalization technology [31] is utilized for ECG signals to map the amplitudes ranging from 0 to 1.

#### 2.2.3. Extraction of Ground Truth BP Values

As we know well, BP values can be extracted from the synchronized ABP signals. SBP ($Y^{s}$) and DBP ($Y^{d}$) values are obtained by computing the maximum and minimum value of a segmented ABP signal, respectively. Additionally, MAP ($Y^{m}$) value is derived from SBP and DBP values, which can be defined as follows:

(7) $$\;Y^{m}=\frac{Y^{s}+2Y^{d}}{3}$$

Meanwhile, the synchronized ABP signals are also with much artifact noise. It leads to incorrect ground truth BP extraction, which would reduce the BP estimation of the proposed method. Skewness, a widely used signal quality assessment tool, is utilized to assess the quality of ABP signals [32]. To be more specific, skewness indexes of 1-s-long ABP segment are calcuated on a 10-s-long ABP segment without overlapping. If any skewness index is less than 0, the entire 10-s-long ABP segment will be discarded, as well as the corresponding synchronized 10-s-long ECG signals.

### 2.3. Proposed Method

**Figure 3:**
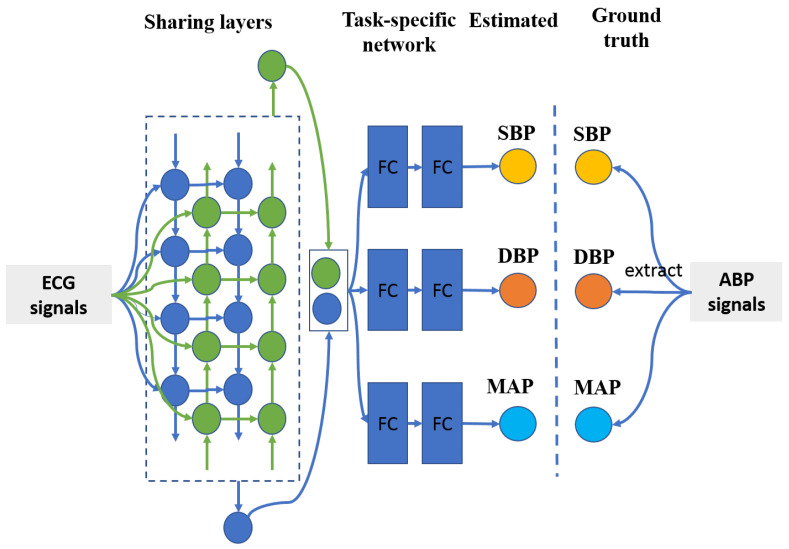
The architecture of the proposed method. It was composed of sharing layers and task-specific networks. FC means fully connected layer.

It is well known that ground truth SBP and DBP values are extracted from an identical ABP segment by computing the maximum and minimum amplitude values, respectively. Therefore, there exists a strong relationship between SBP and DBP values, let alone MAP value, which is derived from SBP and DBP values. Multitask learning has the ability to take advantage of commonality among different tasks to boost the entire classification/estimation performance [33]. Herein, the proposed BP estimation method is based on the multitask deep learning framework, which is comprised of a sharing network and tree task-specific networks, which is shown in Figure 3. To be more specific, a two-layer bidirectional long short-term memory network [34], with the merits of capturing signal information from both forward and backward directions, is employed as the sharing network. As for the task-specific sub-networks, there are three identical two-layer fully connected networks with rectified linear unit (ReLU) [35] as their activation functions to estimate SBP, DBP, and MAP values. The detailed configuration of the proposed method refers to Table 1.

Furthermore, as described in Section 2.1, the proposed multitask deep network utilizes a widely used joint training technique to optimize weighted objective loss function. However, the performance of the multitask network is sensitive to the weights, $\theta^{i\in \{s,d,m\}}$, of task-specific losses. Generally, previous methods tuning weights of task-specific losses are usually grid search and heuristic technologies [26,33]. These kind of methods are extremely time consuming, which often take many days to complete a training trial. In this study, we propose an adaptive weight learning-based method via the estimation loss trend on validation dataset, which is presented in Algorithm 1. Apparently, if the trend of estimation error loss variation is less, it signifies that the optimized space of the task is small. Meanwhile, the mean value and standard deviation of the task-specific estimation error loss are also considered. If they are less on the specified task, it also suggests that there is not much optimal space for it to improve its performance. Herewith, the weight of the task-specific estimation error loss can be defined to be the product of the trend, mean value, and standard deviation of task-specific estimation error. What’s more, all the trend, mean value, and standard deviation of estimation errors are calculated based on validation dataset, which is helpful to improve the generic performance. Specifically, training the proposed multitask network in each iterated epoch can be divided into two phases: training phase and validation phase. In the training phase, the parameters of the proposed network are updated by its forward and backward operation based on error losses formulated in Equation (4). In the validation phase, the trained model is transferred on validation dataset to obtain absolute mean estimation error losses $L^{i\in \left\{s,d,m\right\}}$ for all batches, which can be utilized to compute mean value $L_{mean}^{i}$ and standard deviation $L_{std}^{i}$ of estimation error losses. Based on them, the trend of mean value $T_{mean}^{i}$ and standard deviation $T_{std}^{i}$ of error losses can be formulated by

(8) $$\;T_{mean}^{i}=\frac{{∥L_{mean}^{i}\left(k\right)-L_{mean}^{i}\left(k-1\right)∥}_{1}}{L_{mean}^{i}\left(k\right)}$$

(9) $$\;T_{std}^{i}=\frac{{∥L_{std}^{i}\left(k\right)-L_{std}^{i}\left(k-1\right)∥}_{1}}{L_{std}^{i}\left(k\right)}$$

where *k* is the *k*-th iterated epoch, and *i* is the specified BP estimation task. Suppose that the task with bigger validation trend value has much more importance, the task-specific weight $\theta^{i}$ can be formulated as follows:

(10) $$\;\theta^{i}=\frac{\theta^{i}}{\sum_{i}\theta^{i}},\forall i\in \left\{s,d,m\right\}$$

where $\theta^{i}=T_{mean}^{i}*T_{std}^{i}*\left(L_{mean}^{i}+L_{std}^{i}\right)$. *s* refers to the SBP estimation task, *d* refers to the DBP estimation task, and *m* refers to the MAP estimation task.
**Algorithm 1** Training the proposed adaptive weighted multitask network.**Require:** Training ECG signals $X_{train}^{ECG}$, validation ECG signals $X_{val}^{ECG}$
**Ensure:** Multitask neural network model MODEL to BP estimation
1:Initialize task loss’ weight $\theta^{i}$← 1/32:Initialize multitask network weights including sharing weights $W^{share}$ and task-specific weights $W^{i}$3:Initialize maximum iterated epochs *N*4:**for**k=0→N**do**5:    # Training phase6:    Load $X_{train}^{ECG}$7:    Update parameters of MODEL with loss function of Equation (4)8:    # Validation phase9:    Load $X_{val}^{ECG}$, dividing into *M* batches10:    Load trained MODEL and validate11:    Compute mean value $L_{mean}^{i}\left(k\right)$ and standard deviation $L_{std}^{i}\left(k\right)$ of losses of *M* batches12:    **if** k>0 **then**13:        Compute trend of mean value of losses $T_{mean}^{i}$ based on Equation (8)14:        Compute trend of standard deviation of losses $T_{std}^{i}$ based on Equation (9)15:        Compute task-specific weight $\theta^{i}$ based on Equation (10)16:    **end if**17:**end for**


### 2.4. Reference Methods

Besides the published paper using single-lead ECG signals [17], to the best of our knowledge, to estimate BP values, we also implement popular machine learning methods, such as least absolute shrinkage and selection operator (Lasso) [36], random forests (RF) [37], and support vector regression (SVR) [38], with feature extraction through linear principle component analysis (PCA) [39] and non-linear kernel PCA (KernelPCA) [40] to estimate BP values for comparison.

#### 2.4.1. Feature Extraction Methods

• PCA: PCA is a common method of data analysis, widely used for dimensionality reduction of high-dimensional data. The main idea of the PCA is to transform data features from N-dimensions to K-dimensions. It should be noted that the dimension size of K is far less than than of N. This is a new orthogonal feature, also known as principal component (PC), which is a k-dimensional feature reconstructed on the basis of the original N-dimensional features. To put it simply, PCA is essentially a basis transformation that maximizes the variance of transformed data. In other words, rotation of coordinate axes and translation of coordinate origin minimize the variance between one of the axes (spindle) and data points. After coordinate transformation, orthogonal axes with high variance are removed to obtain dimensionality reduction data set.
• KernelPCA: as is well known for us, KernelPCA is an improved version of the PCA. Compared with the PCA, KernelPCA transforms n-dimensional features to k-dimensional features through kernel technology, which has the capability of transforming data into high-dimensional space through non-linear mapping. Therefore, KernelPCA can transform nonlinear separable data into a new low-dimensional subspace suitable for linear regression of alignment.

#### 2.4.2. Traditional Machine Learning Methods

• Lasso: Lasso is a kind of linear estimation method by importing an additional $l_{1}$ penalty function on the objective function to compress the coefficients in the estimation model. That is, the sum of absolute values of mandatory coefficients is less than a fixed value; Some regression coefficients are set to zero. Thus, subset contraction is retained as a biased estimation for data with complex collinsupport vector regression.
  (11) $$\;\hat{y}=WX+b$$
  where *W* refers to regression weights, *b* refers to regression bias, *X* is a input sample, and $\hat{y}$ is the corresponding output. A widely used method for training Lasso is to minimize the $l_{2}$ loss, following an additional penalty function defined as:
  (12) $$\;minimize{∥\hat{y}-y∥}_{2}+{∥W∥}_{1}$$
• RF: RF is a combinative classifier. Its main idea is to use bootstrap method to randomly re-sample *k* samples from the original data set with the sample size of *N*. Then, a decision tree, as the base classifier of the RF, is utilized to repeatedly build models for new generated data sets. Finally, these decision tree models are combined together to obtain the final classification results by major voting technique. The RF has better classification performance and noise tolerance through random sampling, tree building, and the collection of multiple trees. It has been widely used in all walks of research domains and achieved comparative results.
• SVR: SVR is an important branch of support vector machine (SVM). Inheriting the principle of SVM, SVR is also searching a regression hyper-plane and making all the data in a training dataset closest to that plane. The principle of SVR determines that SVR can achieve a rather good trade-off between the empirical error and complexity during training phase [41]. What’s more, SVR maps the input data into a higher dimension space by the kernel technology. Linear kernel, polynomial kernel, and gaussian radial basis function are common used kernels in applications of SVM. In this study, we employ the RBF kernel due to its capability of high non-linear transformation, since RBF kernel can map input data from low dimension space into a infinite dimension space with Gaussian function.

### 2.5. Deep Learning Methods

Apart from comparison with traditional machine learning methods, the Res2Net [42] following three two-layer fully connected sub-neural networks, which is a representative state-of-the-art deep learning network architecture, is implemented for BP measurement. The Res2Net has the ability to achieve the multi-scale representation of fine-grained level by classifying the residual connections in a single residual block, and at the same time, improve the size of the receiver field in each layer of the network. In this study, specifically, three models of Res2Net50, Res2Net101, and Res2Net152 are employed to capture the multi-scale morphological feature information from ECG signals, the network architectures of which are kept the same as [42], except convolutional filter kernels. The convolutional filter kernels that this study used are one-dimensional, the size of which are set to be the same with [42] as well. The configuration of three full connected neural networks connected by Res2Net are identical to the proposed BP measurement method.

### 2.6. Performance Metrics

Mean error (MErr), mean absolute error (MAErr), and root square mean error (RSMErr) are commonly used measurement tools for evaluating BP estimation models. These measurement tools are formulated as follows:

(13) $$\;MErr=\frac{\sum_{i=1}^{N}\left(y_{i}-{\hat{y}}_{i}\right)}{N}$$

(14) $$\;MAErr=\frac{\sum_{i=1}^{N}\left|y_{i}-{\hat{y}}_{i}\right|}{N}$$

(15) $$\;RMSErr=\sqrt{\frac{\sum_{i=1}^{N}{\left(y_{i}-{\hat{y}}_{i}\right)}^{2}}{N}}$$

where *N* refers to the number of input data, *y* refers to the ground truth BP values, and $\hat{y}$ is the BP value by the estimation model.

What’s more, the BHS and AAMI standards are more widely used measurement tools to evaluate the performance of BP estimation models. A BP estimation method can pass the AAMI standards when the MErr and MAErr are less than 5 mmHg and 8 mmHg, respectively. Otherwise, the BP estimation method fails to pass the AAMI standard. As for the BHS standard, a BP estimation method is graded as Level A, B, or C, by computing the cumulative percentage (CP) of MErrs on the test dataset failing within ±5 mmHg, ±10 mmHg, and ±15 mmHg. In addition, the Bland–Altman method [43] is employed as well for evaluating the difference between the ground truth BP values and estimated BP values.

### 2.7. Data Source

Physionet (https://physionet.org/, accessed on 25 December 2020) freely provides huge numbers of clinical and physiological signals for researchers. One of them, the Physionet Multiparameter Intelligent Monitoring in Intensive Care (MIMIC II Version 3.0) Waveform Database [44], which includes ECG signals and synchronized ABP signals, is usually utilized for researching cuffless BP estimation methods. In this study, we also utilize this waveform database for our proposed method’s training and evaluation. The MIMIC II database has a total of 21,422 ECG and ABP signals, duration of which are different with each other. They vary highly depending on the choice of data physicians and nurses. ECG and ABP signals are with sampling frequency at 125 Hz. Lead II of ECG signals is selected in this study due to its dominant percentage in the total amount of ECG signals and obvious waveform patterns. Ground truth SBP, DBP, and MAP values can be extracted from ABP signals described in preprocessing Section 2.2.3. Meanwhile, ECG and synchronized ABP signals are randomly divided into three independent datasets of training set, validation set, and testing set, the accounting percentages of which are 80%, 10%, and 10%, respectively.

### 2.8. Computing Environment

In this paper, experiments are performed on a computing server, which installs Ubuntu 16.04.6 LTS as its operation system. Since the proposed BP estimation method is a deep network, a widely used deep learning framework, Pytorch 1.0.1, is deployed on the computing server. Regarding hardware configuration of the computing server, it equips itself with a 4-core Intel Xeon CPU at 2.90 Hz, 8 pieces of DDR4 memory cards with 8 GB, and two 128-core NVIDIA GP104GL GPU cards at 1.73 Hz. The powerful computing hardware is helpful to accelerate the speed of all experiments.

## 3. Results

### 3.1. Estimation Performance

In this study, to improve the convergence speed of the proposed BP measurement model, all learned parameters of the weights and biases should be initialized with very small random non-zero floating point values, which are from a uniform distribution of $U(-\sqrt{d},\sqrt{d})$ utilized, where *d* is the hidden size of 256. The entire BP measurement model is optimized with the mini-batch SGD optimizer with momentum (momentum-SGD optimizer) in the training phase, which has the merits of speeding the convergence on big dataset and depressing fluctuation of error losses [45]. At the beginning, the momentum-SGD optimizer in this work uses a learning rate of 0.0008, mini-batch size of 128, and momentum of 0.9. Other parameters of the momentum-SGD optimizer are keeping default values defined by the Pytorch framework. Furthermore, when it is found that the training error loss no longer decreases, the learning rate is reduced with a learning scheme of ReduceLROnPlateau (https://pytorch.org/docs/stable/optim.html, accessed on 25 December 2020) with a factor of 0.9 and patience of 4, other parameters are kept by default values. In order to improve the generalization ability of BP measurement performance, a penalty term of the L2 loss with a factor of 0.1 are employed on the all the weights and biases of the proposed BP model. A total of 500 epochs are set for the BP measurement model.

**Figure 4:**
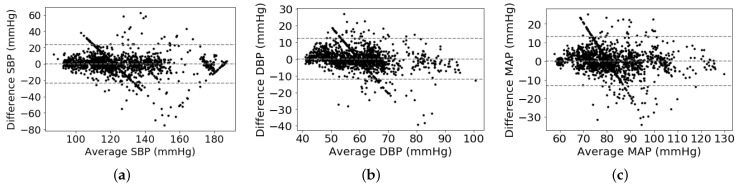
Bland–Altman analysis plots: (**a**) SBP, (**b**) DBP, (**c**) MAP.

**Figure 5:**
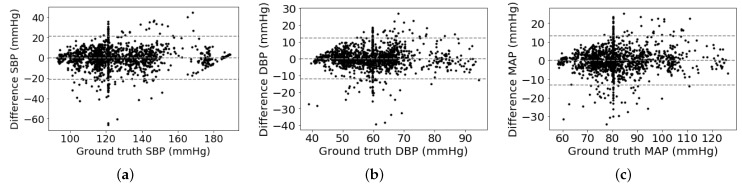
Variant Bland–Altman analysis plots: (**a**) systolic blood pressure (SBP), (**b**) diastolic blood pressure (DBP), (**c**) mean arterial pressure (MAP).

As shown in Table 2, we can observe that the proposed multitask network with the weight-learning scheme obtains a promising BP estimation results. To be more specific, our proposed method obtains performance of 0.12 ± 10.83 mmHg, 0.13 ± 5.90 mmHg, and 0.08 ± 6.47 mmHg for estimating SBP, DPB, and MAP values, respectively. The proposed method with promising BP estimation results can pass the AAMI standard and obtain a quite good grade defined by the BHS standard. In addition, Bland-Altman plot is employed to evaluate BP measurement performance of the proposed method, which presents in Figure 4. It can be observed that most of the estimation points of SBP, DBP, and MAP are falling within the limits of 0.18 ± 21.22 mmHg, 0.13 ± 11.56 mmHg, and 0.08 ± 12.68 mmHg, respectively. It indicates that the proposed BP estimation method has the ability to regress BP values using ECG signals. On the other hand, it seems that some points in the Bland–Altman plot have a linear relationship among them. The cause is that many ground truth BP values are to be around a fixed value. For instance, the fixed values of SBP, DBP, and MAP are around 120 mmHg, 60 mmHg, and 80 mmHg, respectively. According to the principle of the Bland–Altman plot, its x-axis is the average BP values of estimated and ground truth BP values and its y-axis is the difference between estimated and ground truth BP values. Therefore, these points of differential BP values are varying along y-axis by around a fixed point at x-axis, which is shown in a variant Bland–Altman plot in Figure 5. The variant Bland–Altman plot has ground truth BP values as its x-axis instead of mean values of estimated and ground truth BP values.

### 3.2. Performance Comparison

In this section, the proposed method is compared with various BP measurement methods, like the BiLSTM based single task method, BiLSTM based multitask method without weight-learning scheme, reference and cutting-edge BP measurement methods, which are described in detail as follows.

#### 3.2.1. Compared with Single Task Method and Multitask Method without Weighting Scheme

In this study, to verify whether using a multitask scheme has the ability to boost its BP measurement performance or not, a single task based two-layer BiLSTM is implemented to measure BP values. The network configuration of the BiLSTM is set to be the same as the sharing layer in the proposed network. The BP values are estimated by the BiLSTM by concatenating the two ending output units from two opposite directions in the last layer. What’s more, we train the backbone multitask network as the multitask method for comparison. The backbone network also takes advantage of the joint training technology, like the way in which the proposed method employs but with uniform task-specific weights. Here, all the task-specific weights are assigned to be 1. As shown in Table 3, it can be observed that the multitask method has achieved a considerable margin of BP estimation performance to the BiLSTM-based single task method. It demonstrates that there exists close relationship among SBP, DBP, and MAP estimation tasks, which is helpful to boost BP estimation performance with the multitask method. Meanwhile, it is also noted that the proposed method obtains much better BP estimation performance than the multitask method under identical training configurations. It indicates that the weighting scheme can enable the multitask network to improve its BP estimation performance.

#### 3.2.2. Compared with Reference and Cutting-Edge Deep Learning Methods

In this section, we implement popular machine learning regression methods, such as Lasso, RF, and SVR. To reduce the dimension of ECG signals, PCA and KernelPCA are employed to extract key features from ECG signals. Both reference regression methods and feature extracting methods are described in detail in Section 2.4. Hyperparameters of the reference methods are fine-tuning with the grid-search technology. Extensive experiment results are shown in Table 4. One can observe that in most cases, machine learning methods with feature extraction by KernelPCA have much better BP estimation performance. The cause may be that key features existing in ECG signals are non-linear and KernelPCA with RBF kernel has the capability to extract non-linear information. Compared with reference BP estimation methods, our proposed method has achieved promising BP estimation results with a considerable margin.

Furthermore, the proposed method is also compared with deep learning based state-of-the-art BP measurement methods using ECG signals [16,21,25]. Besides, we implement a state-of-the-art deep neural network namely Res2Net [42], with few necessary modifications described in Section 2.5, to estimate BP values. As shown in Table 5, it is noted that our proposed BP measurement method is superior to the deep learning-based BP measurement methods of [16,42], but little inferior to the BP measurement methods of [21,25]. All of the BP measurement methods of [21,25], and our proposed can meet the AAMI standard and BHS standard. Compared with the BP measurement method [21], using the particle swarm optimization (PSO) scheme searching the optimal task-specific importance, the proposed method can compute the task-specific importance directly based on an adaptive weight learning-based scheme, with the merit of requiring much less training time. Even if the performance of the BP measurement method proposed by Miao et al. [25] seems much better than ours, their proposed BP method requires additional patients’ previous BP values to calibrate, while our proposed BP method is calibration-free.

## 4. Discussion

### 4.1. Difference of BP Measurement Performance

**Figure 6:**
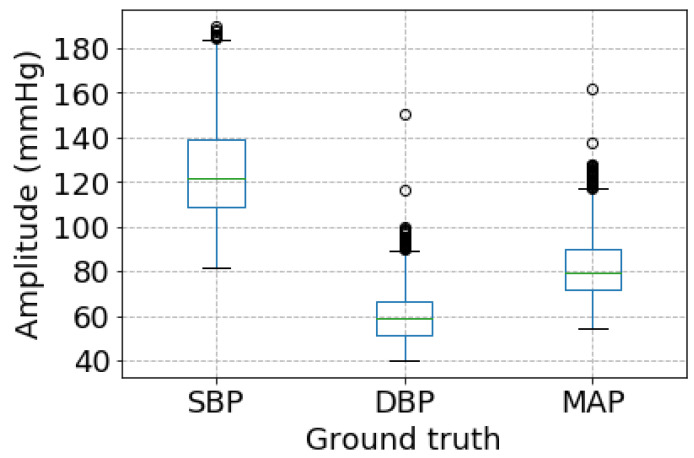
The boxplot of ground truth BP values.

As shown in Table 2, it is not difficult to observe that there are greatly different performances among SBP, DBP, and MAP measurement. DBP estimation achieves the best performance while SBP estimation performance is the worst. The reason is likely to be that the proposed BP measurement method is sensitive to the range of a specified BP values. More specifically, as shown in Figure 6, BP estimated ranges are much different among SBP, DBP, and MAP. SBP has the biggest BP estimated range, followed by MAP, and DBP has the smallest one. It demonstrates that there exists a close relationship between the BP estimation performance and BP estimated range.

### 4.2. Model Parameters Tuning

The selection of number of layers in the BiLSTM, which is a quite important hyper parameter in the proposed method, is discussed. As well known to us, the number of layers of BiLSTM has much more impact on its performance. Herein, the numbers of BiLSTM layers are set to be commonly used numbers; 1, 2, and 4. As shown in Table 6, it is noted that the backbone network of the proposed method, which is a multitask network without a weighting scheme, achieves its best BP estimation performance when the number of BiLSTM layers is set to be 2. Therefore, the proposed method selects a two-layer BiLSTM as its sharing network.

Furthermore, the penalty factor of L2 is another important hyperparameter of the proposed method that greatly affects the BP measurement performance. In this study, the penalty factor is selected from $10^{-4}$ to 10 with a stride by a factor of 10. As shown in Table 7, it is noted that the proposed method can obtain comparable BP measurement performance when the penalty factor of L2 is selected among the set of 0.001, 0.01, and 0.1. Particularly, as the penalty factor of L2 is selected as 0.1, the proposed method achieves its best MAErr of 7.69 mmHg, 4.36 mmHg, and 4.76 mmHg for SBP, DBP, and MAP measurement, as well as the best RMSErr of 12.30 mmHg and 6.88 mmHg for SBP and DP measurement. The best RMSErr of MAP that the proposed methd achieved is 7.39 mmHg when the penalty factor of L2 is set to 0.01, which surpasses the second optimal RMSErr, the penalty factor L2 of 0.1, with 0.13 mmHg. Both of the top 2 optimal RMSErr fall in the error limits of the AAMI standard. Therefore, the proposed BP method sets the penalty factor of L2, to be 0.1 in this study.

## 5. Conclusions

In this study, a novel calibration-free adaptive weight-learning based multitask deep learning framework via single lead ECG signals is proposed for long-term continuous BP estimation. Specifically, the proposed method utilizes a 2-layer BiSLTM as the sharing network, followed by three 2-layer fully connected networks for task-specific BP estimation. To learn the importance of each task-specific loss automatically, an adaptive weight-learning scheme based on the trend of validation loss is proposed. Extensive experiments on the MIMIC II waveform database demonstrates that the proposed network obtains promising results of 0.18 ± 10.83 mmHg, 0.13 ± 5.90 mmHg, and 0.08 ± 6.47 mmHg on SBP, DBP, and MAP estimation, respectively. The proposed method can pass the AAMI standard and meet the requirements of the BHS standard. Meanwhile, the proposed method is also compared with many popular cutting-edge methods using ECG signals. Our proposed method outperforms them with a considerable margin. Our proposed method can be deployed in a healthcare platform or/medical system to provide a BP-health protected service combined with a wearable/portal ECG device. With the help of the BP-protected service, the malignant complications of hypertension would be greatly reduced.

## Figures and Tables

**Table 1:** Configuration of The Adaptive Weight Learning-based Multitask Network.

Network Component	Network Parameters Description
Sharing network layers	Backbone: BiLSTM
	Bidirectional: true
	Sequence length: 20
	Time steps: 50
	Size of hidden unit: 256
	Number of layers: 2
Task-specific sub-network	Sub-network: FC network
	Number of hidden layers: 2
	Number of hidden units in 1st layer: 512
	Number of hidden units in 2nd layer: 256
	Activation function: ReLU

**Table 2:** Estimation Performance of The Proposed Method.

	Index	SBP	DBP	MAP
The proposed	MErr (mmHg)	0.12	0.13	0.08
	MAErr (mmHg)	7.69	4.36	4.76
AAMI [4]	MErr	≤5 mmHg		
	MAErr	≤8 mmHg		
CP of The proposed	≤5 mmHg	53.05%	71.52%	70.03%
	≤10 mmHg	76.56%	89.56%	88.07%
	≤15 mmHg	86.64%	95.03%	94.12%
	Grade	A	B	C
CP of BHS [7]	≤5 mmHg	60%	50%	40%
	≤10 mmHg	85%	75%	65%
	≤15 mmHg	95%	90%	85%

**Table 3:** BP Estimation Performance for the Proposed Method with Different Neural Network Architectures and Searching Scheme of Task-specific Importance.

Methods	Index	SBP	DBP	MAP
BiLSTM (Single task BP estimation method)	MAErr (mmHg)	9.35	5.25	5.21
	RMSErr (mmHg)	13.88	7.93	9.35
Multitask method (Multitask BP measurement methods without weight searching scheme)	MAErr (mmHg)	8.39	4.49	4.92
	RMSErr (mmHg)	12.77	6.94	7.32
The proposed	MAErr (mmHg)	7.69	4.36	4.76
	RMSErr (mmHg)	10.83	5.90	6.47

**Table 4:** Blood pressure (BP) Estimation Performance Comparison with Reference Machine Learning Methods.

Methods (Feature + BP Model)	Index	SBP	DBP	MAP
PCA + Lasso	MAErr (mmHg)	12.25	9.57	8.59
	RMSErr (mmHg)	15.68	13.21	12.01
PCA + RF	MAErr (mmHg)	12.14	10.08	8.18
	RMSErr (mmHg)	15.66	13.38	11.34
PCA + SVR	MAErr (mmHg)	11.85	8.84	7.27
	RMSErr (mmHg)	15.23	12.59	11.44
KernelPCA + Lasso	MAErr (mmHg)	12.24	8.95	7.98
	RMSErr (mmHg)	15.44	12.52	11.4
KernelPCA + RF	MAErr (mmHg)	14.14	10.00	14.37
	RMSErr (mmHg)	17.51	13.32	11.51
KernelPCA + SVR	MAErr (mmHg)	11.83	8.80	7.26
	RMSErr (mmHg)	15.22	12.58	11.44
The proposed	MAErr (mmHg)	7.69	4.36	4.76
	RMSErr (mmHg)	12.30	6.88	7.52

**Table 5:** BP Estimation Performance Comparison with Deep Learning methods.

Methods	Index	SBP	DBP	MAP
Res2Net50 [42]	MAErr (mmHg)	10.79	6.58	7.50
	RMSErr (mmHg)	14.28	8.18	9.40
Res2Net101 [42]	MAErr (mmHg)	11.99	6.12	7.24
	RMSErr (mmHg)	14.38	7.66	9.09
Res2Net152 [42]	MAErr (mmHg)	10.21	6.63	7.07
	RMSErr (mmHg)	13.45	8.26	8.88
Simjanoska et al. [16]	MAErr (mmHg)	8.64	18.20	13.52
	RMSErr (mmHg)	10.97	19.34	15.07
Fan et al. [21]	MAErr (mmHg)	7.16	3.89	4.24
	RMSErr (mmHg)	10.83	5.90	6.47
Miao et al. [25]	MAErr (mmHg)	7.10	4.61	4.66
	RMSErr (mmHg)	9.99	6.36	6.29
The proposed	MAErr (mmHg)	7.69	4.36	4.76
	RMSErr (mmHg)	12.30	6.88	7.52

**Table 6:** Comparison of the Number of BiLSTM layers in the Backbone Multitask Method.

BiLSTM	Index	SBP	DBP	MAP
1 layer	MAErr (mmHg)	8.87	4.79	5.39
	RMSErr (mmHg)	13.80	7.62	8.36
2 layers	MAErr (mmHg)	8.39	4.49	4.92
	RMSErr (mmHg)	12.77	6.94	7.32
4 layers	MAE (mmHg)	11.64	6.21	6.87
	RMSErr (mmHg)	16.94	8.55	9.97

**Table 7:** BP Measurement Performance of the Proposed Method with Different L2 penalty factors.

L2 Penalty Factor	Index	SBP	DBP	MAP
0.0001	MAErr (mmHg)	8.13	4.11	4.99
	RMSErr (mmHg)	12.74	6.87	8.07
0.001	MAErr (mmHg)	8.04	4.85	4.89
	RMSErr (mmHg)	12.90	7.48	7.61
0.01	MAErr (mmHg)	8.04	4.86	4.97
	RMSErr (mmHg)	12.98	7.08	7.39
0.1	MAErr (mmHg)	7.69	4.36	4.76
	RMSErr (mmHg)	12.30	6.88	7.52
1.0	MAErr (mmHg)	8.65	4.42	5.27
	RMSErr (mmHg)	12.47	7.16	8.05
10	MAErr (mmHg)	11.16	7.31	7.63
	RMSErr (mmHg)	14.60	9.93	10.14

## Data Availability

Publicly available datasets were analyzed in this study. This data can be found here: https://physionet.org/, accessed on 25 December 2020.

## Abbreviations

The following abbreviations are used in this manuscript:

WHO	World Health Organization
BP	blood pressure
SBP	systolic blood pressure
DBP	diastolic blood pressure
MAP	mean arterial pressure
ABP	arterial blood pressure
PWTT	pulse wave transit time
PWAT	pulse wave arrival time
ECG	electrocardiograph
PPG	photoglethysmography
BHS	British Hypertension Society
AAMI	US Association of Advancement of Medical Instrument
PIR	phtopleththysmograph
db8	Daubechies 8
CWT	continuous wavelet transformation
DwT	discrete wavelet transformation
ReLU	rectified linear unit
BiLSTM	bidirectional long short-term memory network
FC	fully connected network
RF	random forest
SVR	support vector regression
PCA	principle component analysis
KernelPCA	kernel PCA
MErr	mean error
MAErr	mean absolute error
RSMErr	root square mean error
CP	cumulative percentage
PSO	particle swarm optimization
